# Quantitative label-free proteomic analysis of human urine to identify novel candidate protein biomarkers for schistosomiasis

**DOI:** 10.1371/journal.pntd.0006045

**Published:** 2017-11-08

**Authors:** Olugbenga Samson Onile, Bridget Calder, Nelson C. Soares, Chiaka I. Anumudu, Jonathan M. Blackburn

**Affiliations:** 1 Biotechnology Programme, Department of Biological Sciences, Elizade University, Ilara-Mokin, Nigeria; 2 Division of Chemical & Systems Biology, Department of Integrative Biomedical Sciences, University of Cape Town, Cape Town, South Africa; 3 Cellular Parasitology Programme, Department of Zoology, University of Ibadan, Ibadan, Nigeria; 4 Institute of Infectious Disease & Molecular Medicine, University of Cape Town, Cape Town, South Africa; Walter and Eliza Hall Institute of Medical Research, AUSTRALIA

## Abstract

**Background:**

Schistosomiasis is a chronic neglected tropical disease that is characterized by continued inflammatory challenges to the exposed population and it has been established as a possible risk factor in the aetiology of bladder cancer. Improved diagnosis of schistosomiasis and its associated pathology is possible through mass spectrometry to identify biomarkers among the infected population, which will influence early detection of the disease and its subtle morbidity.

**Methodology:**

A high-throughput proteomic approach was used to analyse human urine samples for 49 volunteers from Eggua, a schistosomiasis endemic community in South-West, Nigeria. The individuals were previously screened for *Schistosoma haematobium* and structural bladder pathologies via microscopy and ultrasonography respectively. Samples were categorised into schistosomiasis, schistosomiasis with bladder pathology, bladder pathology, and a normal healthy control group. These samples were analysed to identify potential protein biomarkers.

**Results:**

A total of 1306 proteins and 9701 unique peptides were observed in this study (FDR = 0.01). Fifty-four human proteins were found to be potential biomarkers for schistosomiasis and bladder pathologies due to schistosomiasis by label-free quantitative comparison between groups. Thirty-six (36) parasite-derived potential biomarkers were also identified, which include some existing putative schistosomiasis biomarkers that have been previously reported. Some of these proteins include Elongation factor 1 alpha, phosphopyruvate hydratase, histone H4 and heat shock proteins (HSP 60, HSP 70).

**Conclusion:**

These findings provide an in-depth analysis of potential schistosoma and human host protein biomarkers for diagnosis of chronic schistosomiasis caused by *Schistosoma haematobium* and its pathogenesis.

## Author summary

Schistosomiasis, caused by *S*. *haematobium*, causes inflammation in the bladder and is common in tropical areas such as Nigeria. Undetected schistosomiasis can lead to inflammation in the bladder which may lead to bladder cancer. Diagnosis of bladder cancer in areas with common urinary schistosomiasis is difficult because the two diseases share many common symptoms. It is therefore important to identify biomarkers that could be used for early diagnosis of both schistosomiasis and schistosomiasis-associated bladder cancer. We chose a proteomic approach to identify these candidate biomarkers in a clinical cohort of urine samples obtained in Nigeria. We found several parasite- and host-specific protein biomarkers that could have diagnostic potential in a urine-based test for schistosomiasis, and schistosomiasis-associated bladder cancer. These include proteins that may also have application in drug or vaccine development. This study is one of the first to catalogue the urinary proteomes of people affected by this neglected tropical disease.

## Introduction

Urinary schistosomiasis, caused by the parasite *Schistosoma haematobium*, is of public health significance in tropical and sub-tropical areas, with an estimated 732 million persons being vulnerable to infection worldwide in well-defined transmission areas [1]. In 2008, 17.5 million people were treated globally for schistosomiasis, 11.7 million of those from sub-Saharan Africa [2]. Schistosomiasis is considered to be endemic in Nigeria [1,3,4], where about 20 million people are affected by chronic schistosomiasis [1,5]. *Schistosoma haematobium* infection is reported to be more widespread than *Schistosoma mansoni* infection [6]. Schistosomiasis is characterized by continued health threat and inflammatory challenges in people who are exposed to long-term daily risk of infection [7]. Chronic infection with *S*. *haematobium* has been reported as a possible risk factor in the aetiology of bladder cancer [8,9]. Several studies have recorded increased urinary tract pathology conditions among populations infected with *S*. *haematobium* [3,10]. Histopathologists have also associated *S*. *haematobium* infection with the development of squamous cell carcinoma of the bladder [11]. *S*. *haematobium* has been associated with a two- to ten-fold increase in the risk of bladder squamous cell carcinoma, as well as being a potential cause of kidney damage. Hence, the parasite is considered as a group 1 carcinogen [12]. In some regions where *S*. *haematobium* is endemic, bladder cancer is the most common cancer in men and the second most common in women, just behind breast cancer, accounting for as much as 30% of all cancer cases [13].

Early disease detection of bladder cancer would significantly benefit people living in *S*. *haematobium*-endemic areas, because bladder cancer is otherwise unlikely to be recognized, as the obvious urinary tract symptoms (intermittent haematuria, dysuria, increased frequency, urgency and pain with micturition) are so commonly associated with urinary schistosomiasis that when the cancer manifests the patient is not likely to receive adequate diagnosis and may become severely debilitated with poor disease prognosis [10].

Detecting bladder cancer at the population level is challenging because direct proof requires detailed histopathological study, but invasive examinations are restricted to tertiary hospitals [14]. The detection of cancer-associated biomarkers, preferably isolated from urine and blood, has therefore become important. Such biomarkers are now being developed and will provide tools that could be useful to evaluate the specific effects of long-term exposure to *S*. *haematobium* [15].

Demonstration of schistosome-associated bladder damage by ultrasound examination is valuable and useful; however, it cannot be used to construe a diagnosis of cancer. Cancer-specific urine biomarkers may therefore play an important role in people with long-term *S*. *haematobium* infections. In addition, considering the fact that treatment of schistosomiasis relies on a single drug, praziquantel, which raises fears of resistance, there is a need to acquire a deeper understanding of the communication between the parasite and the mammalian host, with a view to identifying new methods of controlling schistosomiasis and schistosomiasis-associated bladder cancer.

One potential approach to investigating the developing relationship between the parasite and its host is proteomics. Biological fluids are promising sources of diagnostic, prognostic and treatment based biomarkers, due to their easy accessibility [16, 17]. Biological fluids are associated with tissues that release protein components into them and the disease-altered state could change either the constituents or the amount of such proteins. Biofluid-derived proteins could be parasite or host associated biomarkers.

Proteomics has been successfully employed for human-based studies of disease, where it has been a valuable approach for distinguishing diseases and generating candidate biomarkers to determine pathological state [16, 18]. Mass spectrometry (MS) based analysis of a small number of exposed and unexposed subjects has been found to reveal altered expression of proteins that may be identified as intermediate biomarkers of early disease effects [19]. In particular, the potential of the urinary proteome as a non-invasive means to identify biomarkers for carcinogen exposure and metabolism of toxic chemicals has been demonstrated by Moore *et al*. [19].

Several schistosome-oriented proteomics studies have focused on the parasites [12,16]. However, more information on the changes that manifest in the host proteome during active schistosomiasis is required [14]. The goal of the present study is thus to identify candidate biomarkers for the diagnosis of schistosomiasis and schistosomiasis-associated bladder cancer from adults in a rural population in south-west Nigeria, an area which is endemic for urinary schistosomiasis.

## Materials and methods

### Urine sample collection

**Fig 1:**
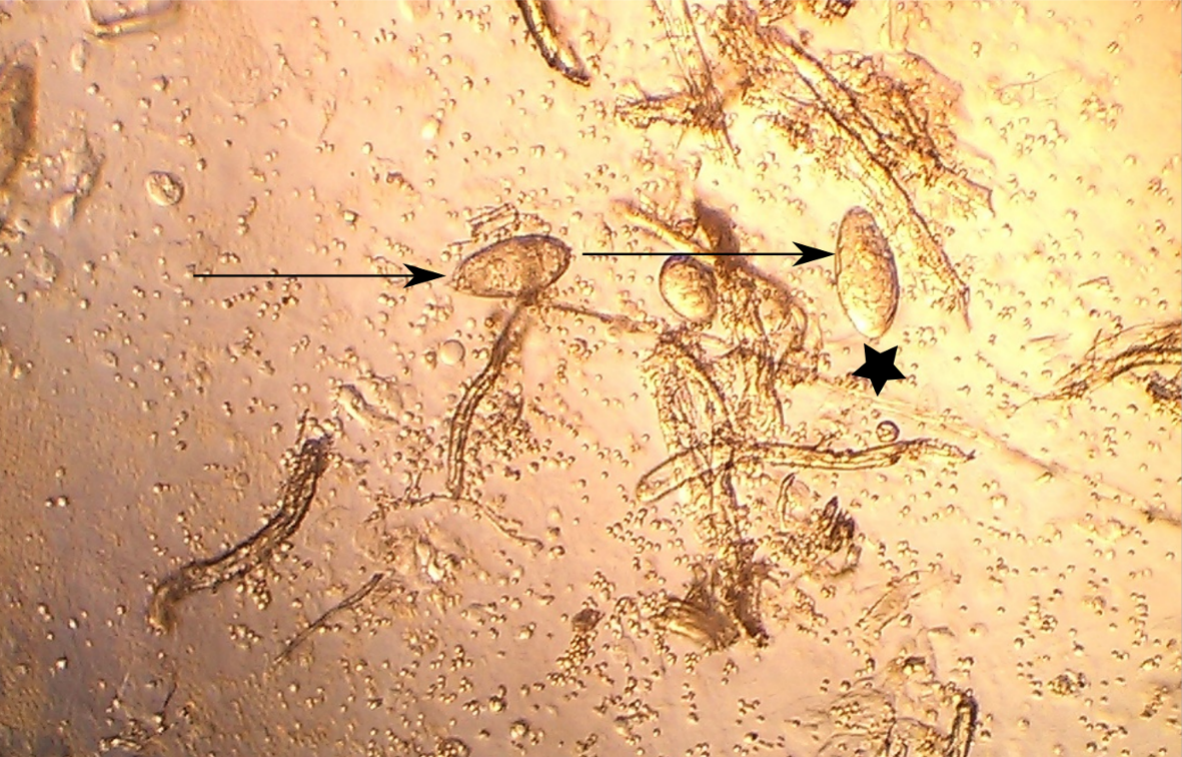
Schistosome eggs (arrows) in the urine of an *S*. *haematobium* infected participant as shown by light microscopy (magnification x10). A terminal spine of the egg is indicated by a black star.

Human urine samples were collected from volunteers living in Eggua, Ogun State, a schistosomiasis endemic community in South-west, Nigeria. Eggua lies between latitude 7° 6ʹ4.811ʺ N and longitude 2° 52ʹ 43.776ʺ E in a derived savanna zone. The area is largely dominated by Yoruba speaking people. The volunteers were screened for the presence of *Schistosoma haematobium* infection by a combination of microscopy (Fig 1), detection of macro and microhaematuria (urinalysis) and rapid diagnostic test (RDT) for schistosomiasis (Table 1). The urine samples were collected between 10:00 and 14:00 to ensure maximum egg yield and 10 mL of sample were processed for microscopic examination and egg count. The eggs were quantified by counting and classified as light infection if there were ≤50 (1–49) eggs/10 mL urine and heavy infection if there were >50 eggs/10 mL urine [10] and urinary structural bladder pathology was examined using ultrasonography, which has been published elsewhere [10]. Sample size power calculation was carried out, indicating that 44 individual samples (*N*) were required for a statistical power of 0.9 at significance level 0.05.

**Table 1:** Pathology and infection status of sampled volunteers.

Sample Group	Gender (n = 49)		*S*. *haematobium* Infection by egg count	Microhaematuria	Infection by RDT	Bladder Pathology
PS	Male	8	Present	Absent	Absent	Present
	Female	7				
SH	Male	5	Present	Present	Present	Absent
	Female	7				
PT	Male	5	Absent	Absent	Absent	Present
	Female	7				
NPS	Male	4	Absent	Absent	Absent	Absent
	Female	6				

### Ethical consideration

Ethical approval was obtained from the University of Ibadan and University College Hospital (UI/UCH) Ethical Committee and Ogun State Ministry of Health. All participants gave informed consent; all participants were adults and were able to decide for themselves. The informed consent document was written in both English and Yoruba languages, the latter being the language of the Nigerian communities. For those participants who could read and write, written informed consent was obtained. For those participants who could not read and write, the informed consent form was read to them in their language. All participants enrolled in the study voluntarily. The informed consent was signed by all participants and those who could not sign provided a thumb print on the informed consent form. This approach to informed consent for those who could not read and write was approved by the UI/UCH Ethical Committee.

### Sample preparation and in solution protein digestion

A total of 49 individual urine samples were placed into four different categories, namely 12 schistosomiasis cases (SH), 12 bladder pathology cases (PT), 15 combined pathology and schistosomiasis (PS) cases and 10 controls with no pathology or schistosomiasis (NPS). All samples were processed on the same day using the same batches of all reagents, including trypsin, to minimise batch effects and sample to sample technical variation. An aliquot of 4 ml of urine per individual was subjected to methanol-chloroform protein precipitation followed by in solution tryptic digest prior to MS analysis. Precipitated protein was resuspended in denaturation buffer (6 M urea, 2 M thiourea, 10 mM Tris buffer, pH 8.0), and then a Bradford assay was carried out to determine protein concentration [17]. For each sample, 100 μg of protein was then further reduced by incubation at room temperature for 1 hour in reduction buffer (1 M dithiothreitol (DTT); 50 mM ammonium bicarbonate (ABC). An alkylating buffer (550 mM iodoacetamide (IAA) in 50 mM ABC) was then added and incubated in the dark at room temperature for an hour. The sample was then diluted with 4 volumes of 50 mM ABC and proteolysed overnight for 16 hours at 37°C using Trypsin-Ultra (mass spectrometry grade; New England BioLabs) according to the manufacturer’s instructions. An equivalent of 10 μg of the peptide solution was then transferred to in-house prepared stage tips for off-line solid-phase extraction, desalting and clean-up of sample, as described in previous studies [17, 20], and the desalted peptides were then dried in a refrigerated speedy vac (SPD 111v-230 Speed VAC, Thermo Savant, New York, USA).

### Ultra-high performance liquid chromatography

Peptide samples were resuspended by diluting the desalted, dried peptides to 200 ng/μL using 2% acetonitrile (ACN) in HPLC grade water containing 0.1% v/v formic acid (FA) before MS analysis. Nanoflow ultra-HPLC was carried out on each sample, without pre-fractionation, using a Dionex UltiMate 3500RSnano UPLC system (Thermo Fisher, San Jose, CA, USA) equipped with a reverse phase (RP) pre-column trap (100 μm × 2 cm; 5 μm; 100 Å; C18) and analytical column (70 μm × 20 cm; 5 μm; 100 Å; C18). Equal 400 ng injections of each sample were eluted by gradient chromatography at 23°C with a flow rate of 300 nL/min, and a 6–40% gradient of water–ACN from 0 to 120 min. The binary mobile phase system used was as follows: buffer A contained water and 0.1% FA, while buffer B contained ACN and 0.1% FA. The elution gradient for peptides was 6% B from 10 min to 40% B at 60 min, then increasing to 80% B for 10 min before returning to 2% B for equilibration. The same pre-column trap and analytical column was used for all samples.

### Mass Spectrometry (MS)

Discovery proteomic analysis of each sample was carried out on a Q Exactive hybrid quadrupole-Orbitrap mass spectrometer (Thermo Fisher). Analysis of samples introduced from the in-line HPLC system was achieved with the following system settings: Data-dependent automated full scan cycles were performed with automatic switching between MS/MS and MS scans at a scan range of 300–1650 *m/z*. The top ten most abundant precursor ions selected by the quadrupole during the initial MS scan were subjected to fragmentation using high-energy collision dissociation with normalized collision energy at a pressure of 1.2 mTorr and a dynamic exclusion time of 30 s. The abundance threshold for ion selection was 0.001 with charge exclusion of *z* = 1 ions. Acquisition of mass spectra was done at a resolution of 70,000 with a maximum injection time of 250 ms or a target automatic gain control value of 3×10^6^. High-energy collision dissociation and normalized collision energy set at 27 were used for peptide fragmentation. Continuous tandem mass spectra acquisition resolution was set at 17,500 at a maximum injection time of 120 ms or target AGC of 2 × 10^5^. The mass spectrometry proteomics data have been deposited to the ProteomeXchange Consortium via the PRIDE partner repository with the dataset identifier PXD006438.

### MS data processing and statistical analysis

All raw MS data was processed with MaxQuant software (version 1.5.3.12) and its built-in search engine, Andromeda, for peptide identification and protein inference, using the default settings and the human and *S*. *mansoni* databases (www.uniprot.org), as described in detail elsewhere [17] (S1 Fig). FDRs were set at 1% at both peptide and protein level. Peptide identifications were transferred to unidentified features in other LC-MS runs based on matching masses and re-calibrated retention times between runs (“match between runs” option in MaxQuant). Normalisation of data between LC-MS runs was carried out in MaxQuant, based on summation of the total peptide ion signals per sample and using a global Levenberg-Marquardt optimisation procedure that aimed to minimise the overall changes for all peptides across all samples [21]. Label-free quantification (LFQ) was then carried out using MaxQuant, based on determination of the median pair-wise common peptide ratios between samples, requiring a minimum of two peptide ratios per identified protein [21].

The LFQ values from MaxQuant were imported into Perseus software (version 1.5.3.1) for differential expression statistical analysis and visualization by hierarchical clustering and Principal Component Analysis (PCA), with a Benjamini-Hochberg multiple testing correction cut off set at FDR 0.05. Three separate independent t-tests were carried out using LFQ data to compare NPS versus SH, NPS versus PT, and NPS versus PS. One-way ANOVA was also carried out to statistically validate the differentially expressed potential biomarkers. Venn diagrams were plotted using VennDIS (version 1.01). Proteins which were determined to be significantly differentially expressed between groups were further subjected to a GO-enrichment analysis using Blast2GO [16,22]. Uniquely identified human proteins were subjected to pathway and protein-protein interaction analysis using the Reactome database (v60; www.reactome.org) and String DB (www.string-db.org), respectively.

## Results

**Fig 2:**
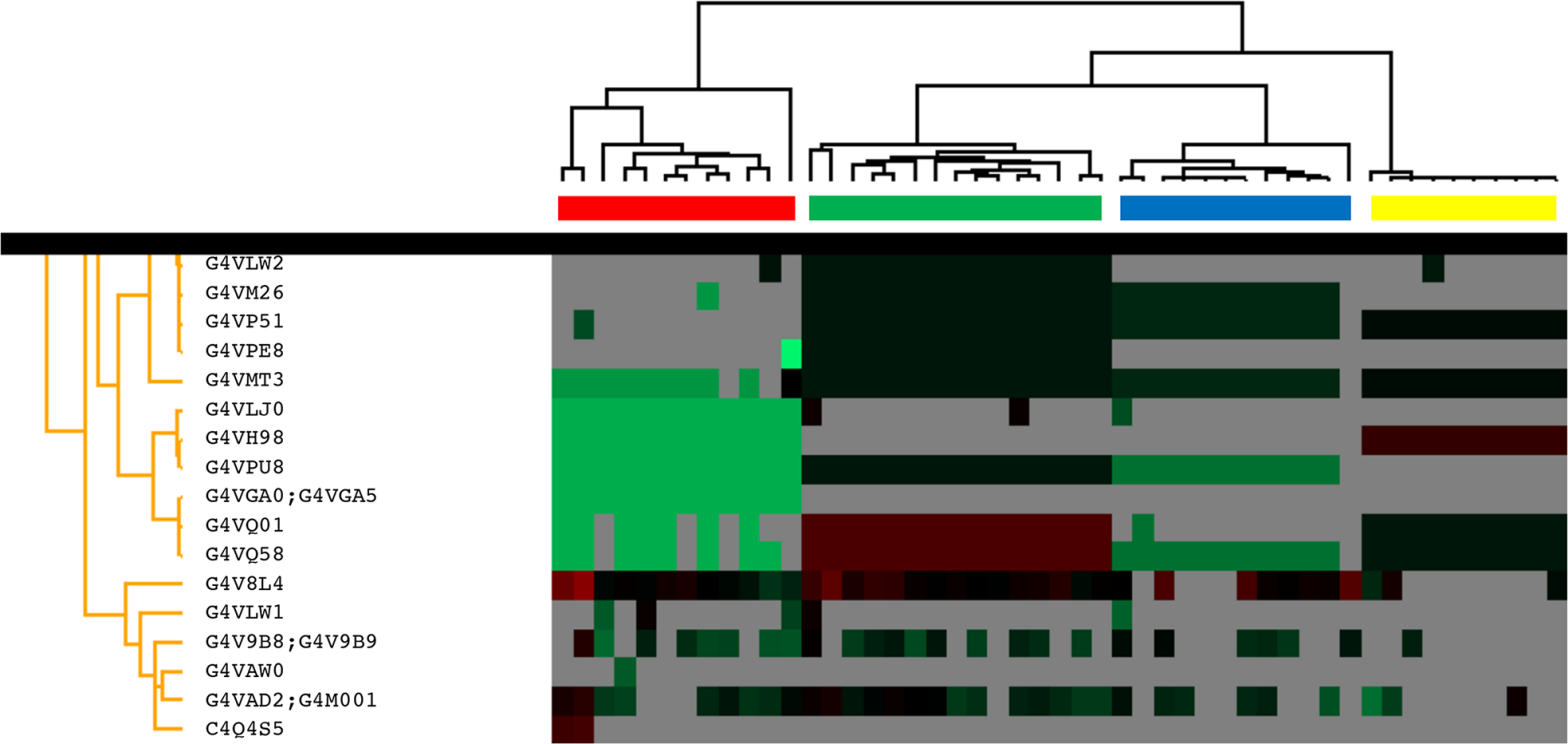
A hierarchical heatmap showing distinct clustering of each sample group. The colour bars above the heatmap depict the following clusters: Schistosomiasis, SH—red; Bladder Pathology, PT—blue; Pathology and Schistosomiasis, PS—green; No Pathology or Schistosomiasis, NPS—yellow. Representative protein identifiers that drive the clustering are also shown.

**Fig 3:**
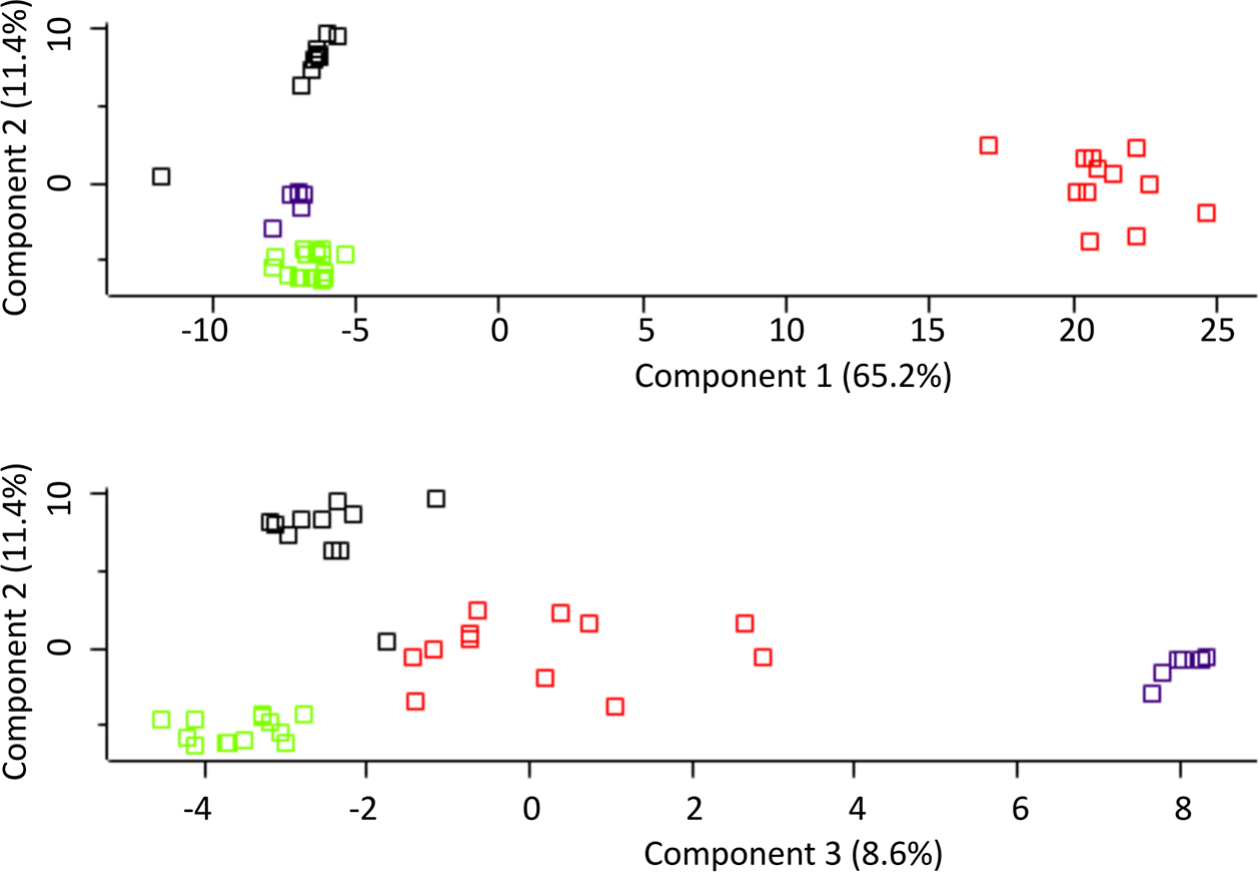
Individual sample comparison between NPS, SH, PS and PT. Similar patterns with minor overlap were seen by multivariate testing using Principal Component Analysis (PCA). The abbreviations represent different sample groups namely: SH—*S*. *haematobium* infected group (red); PT—bladder pathology group (black); PS—group with combination of pathology and *S*. *haematobium* infection (green); and NPS—No Pathology or Schistosomiasis (blue).

Urine samples from 49 individuals distributed across the four disease groups (NPS, SH, PT and PS) were analysed to identify candidate biomarkers for schistosomiasis and its associated pathologies (Table 1). High levels of correlation between the urinary protein components of these sample groups was demonstrated by scatterplots, hierarchical clustering (heatmap) and principal component analysis (PCA) (Figs 2 and 3). The dimensions in Fig 3 account for 76.6% and 20% of the observed variation, respectively. Hierarchical clustering of protein groups identified in SH, PT and PS and NPS samples showed clear molecular differences between groups. As expected, differences in the proteomic signatures were observed between the control group (NPS) and all disease groups, as all three disease groups clustered distinctly, with the SH and PS group being more closely related to one another as compared to the PT group (Figs 2 and 3), although a few samples of the pathology group clustered proximally to the control group (Fig 3).

A total of 209,923 tandem mass spectra were identified and used to assign peptides and unique protein group identities, leading to the identification of 9,701 non-redundant peptides and 1,306 protein groups at a false discovery rate of 1%. The majority (66.3%) of peptides identified had no missed cleavages, while 27.5% had one missed cleavage and 6.2% had two missed cleavages (S4 Fig), which is within the expected range for in solution digest of complex protein mixtures. The number of peptide sequences identified and % MS/MS spectra identified per sample are shown in S2 and S3 Figs, respectively.

Prior to quantitative analysis of differential abundance, samples from each individual were normalised at the peptide level before mass spectrometric analysis. The intensity data for all identified peptides per sample was then further normalised in MaxQuant, based on the underlying assumption that the majority of the proteome should not change significantly between any two conditions and that the average behaviour can therefore be used as a relative standard [21]. In other words, normalisation between samples was based solely on the distribution of the peptide-level data obtained, without addition of external standards or reliance on any set of housekeeping proteins that are assumed to be stably expressed, as is now commonplace in label-free proteomics. Subsequent label-free differential protein abundance analysis was carried out at the protein level using MaxQuant and volcano plots of pairwise comparisons demonstrated the effectiveness of the normalisation procedures, with the majority of proteins found to be not significantly differentially expressed between the various comparisons after permutation-based FDR truncation (FDR = 0.05; S5 Fig).

**Fig 4:**
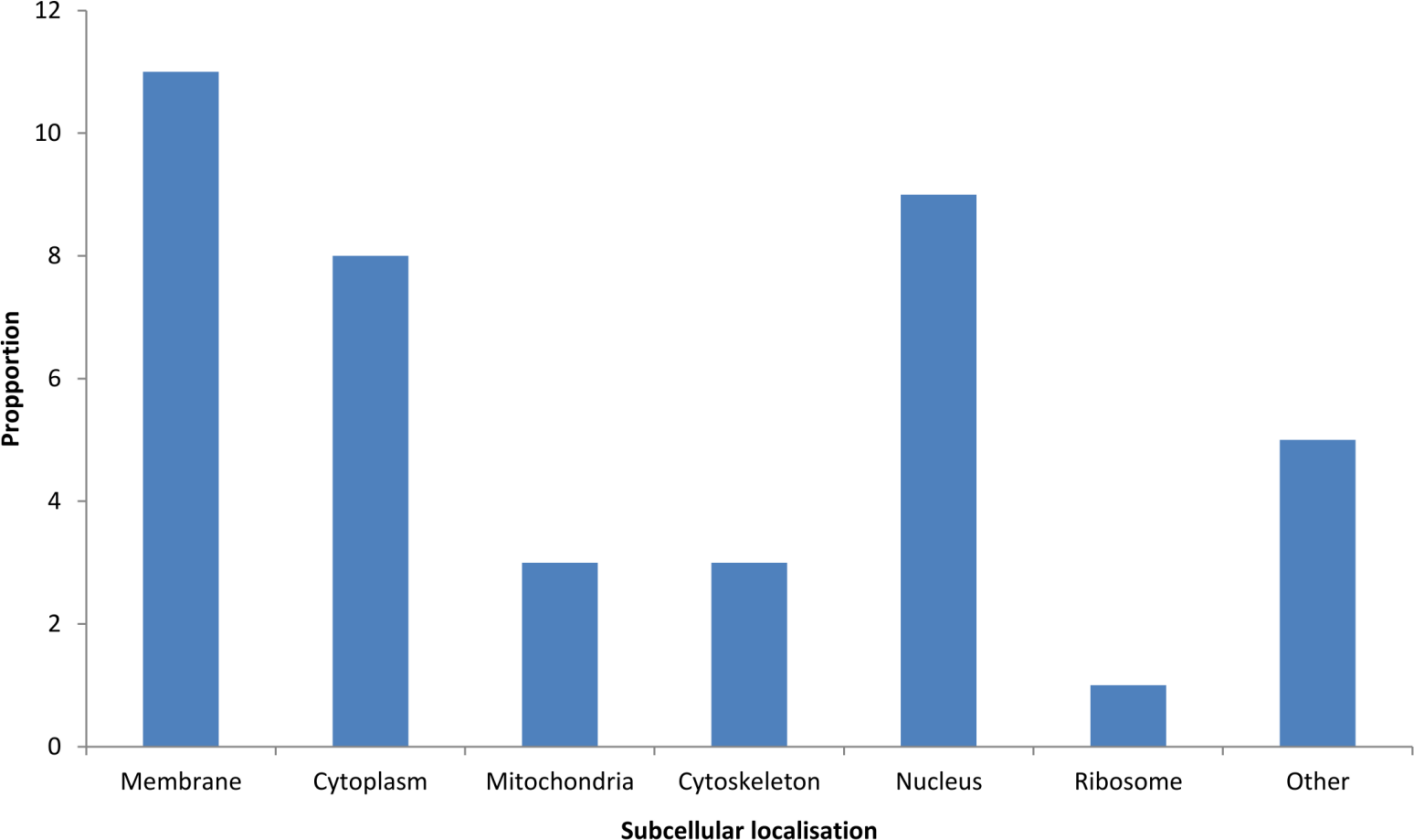
Allocation of observed Schistosoma-derived proteins into various subcellular locations according to Blast2GO analysis.

**Fig 5:**
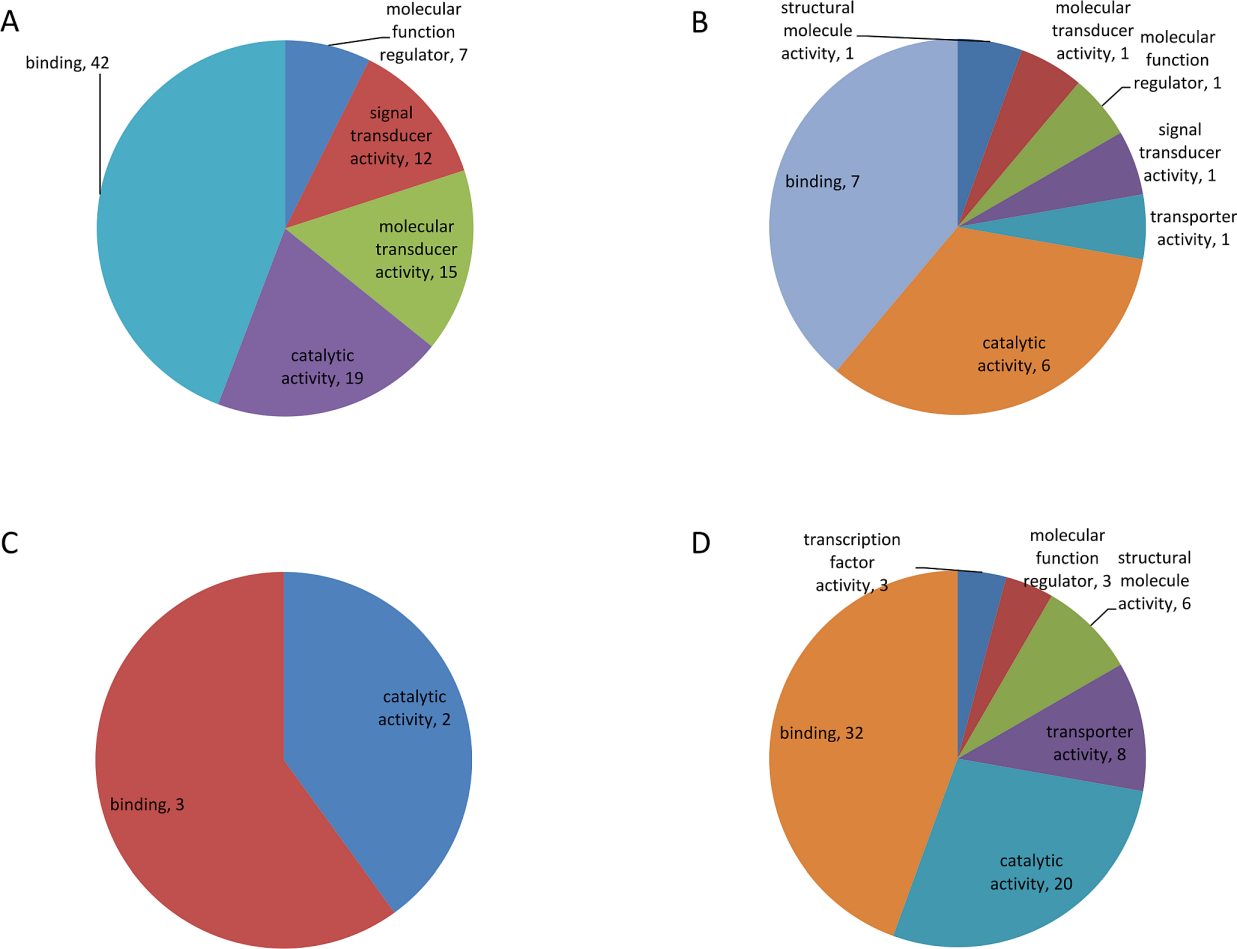
Summarized molecular function of the identified human and Schistosoma proteins as predicted by Blast2GO. The abbreviations represent different sample groups namely: SH—*S*. *haematobium* infected groups (A); PT—bladder pathology group (B); PS—group with combination of pathology and *S*. *haematobium* infection (C); and Schistosoma proteins (D).

**Fig 6:**
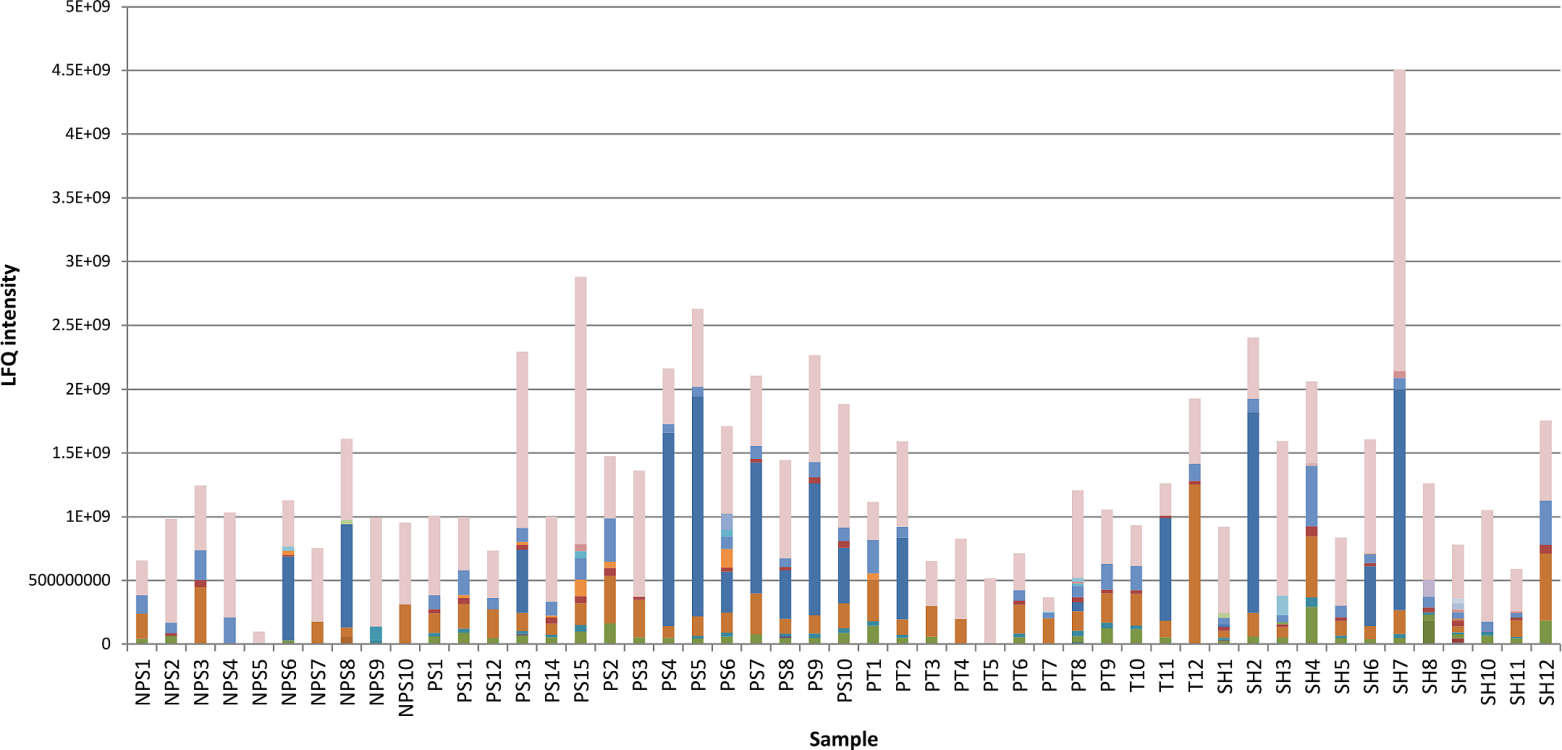
Abundance of Schistosoma-derived proteins and their LFQ intensity among individual samples. SH—Schistosomiasis; PT—Bladder Pathology; PS—Pathology and Schistosomiasis; NPS—No Pathology or Schistosomiasis.

**Fig 7:**
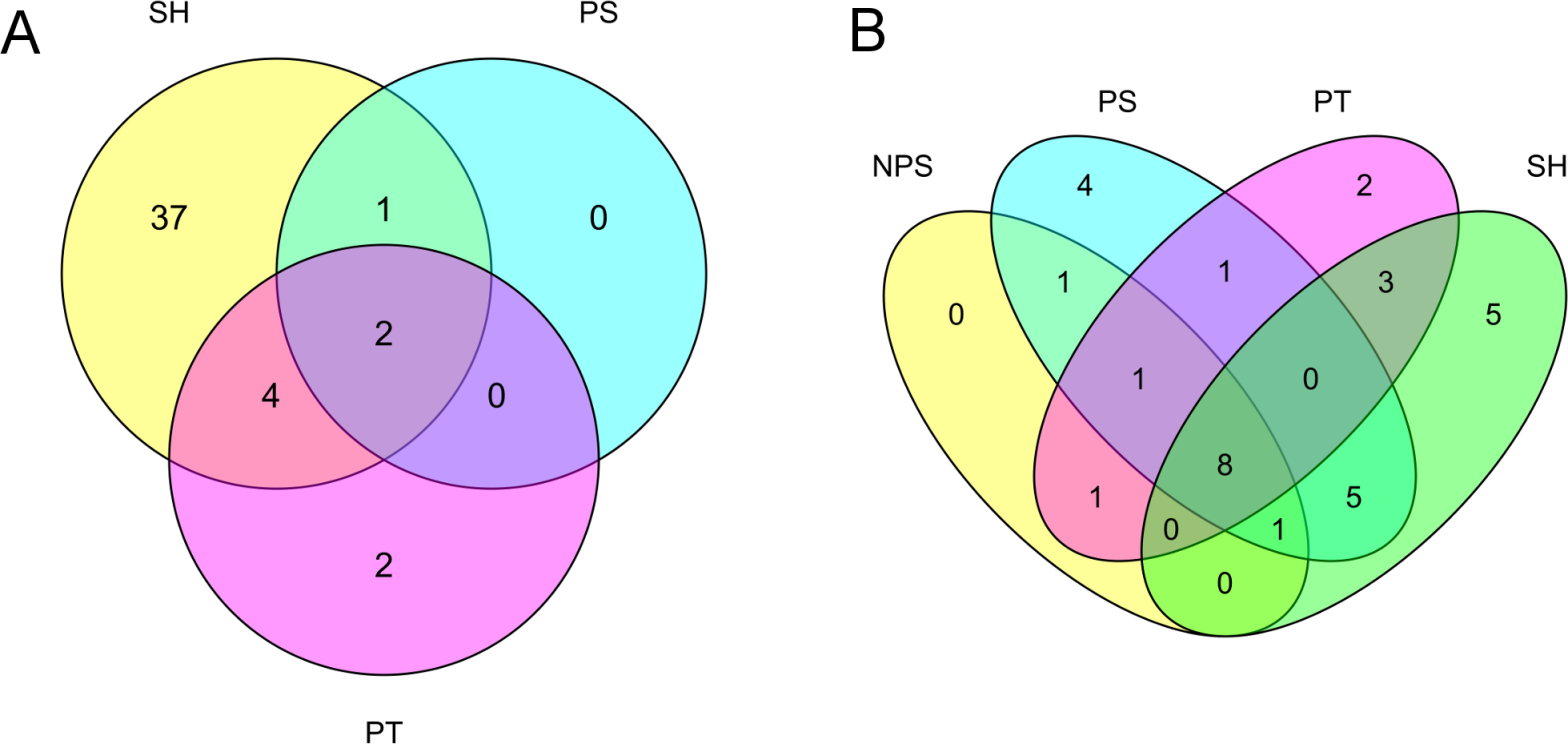
Venn diagrams showing (A) overlap in Schistosoma proteins identified across different clinical groups and (B) overlap in the statistically significant human proteins identified across clinical groups. SH—*S*. *haematobium* infected groups; PT—Bladder Pathology group; PS—group with combination of pathology and *S*. *haematobium* infection; NPS—No Pathology or Schistosomiasis.

A total of 36 *Schistosoma* proteins were identified in the host urine when the MS output was searched against a combination of human and *Schistosoma* databases (Table 2). These 36 identities were considered to be confident identities due to the relatively small size of the *Schistosoma* database compared to the human database in the combined database. The GO terms for the molecular function of the identified human and *Schistosoma-*derived proteins are summarized in Figs 4 and 5. More (124) parasite protein groups were identified when the MS output was searched against the Schistosoma DB only, but only 31 *Schistosoma* proteins were differentially abundant between groups by ANOVA using label-fee quantification (LFQ). Some *Schistosoma* specific proteins were found in samples from individuals earlier diagnosed and classified as negative for *S*. *haematobium* infection by microscopy (Figs 6 and 7).

**Table 2:** Identified Schistosoma-derived proteins across all urine sample groups, and their predicted functions. A posterior error probability (PEP) score cut-off of <0.01 was applied to all protein identities in order to ensure confident protein assignments.

Protein ID	Identified Schistosoma Protein	PEP Score	Location	Predicted Functions
C4Q4S5	Tubulin alpha chain	1.86E-18	Cytoskeleton	Structural/GTPase activity
C4Q5I7	Calreticulin autoantigen homolog	0.00034	Mitochondria	Binding
C4QBN1	Histone H4	0.002522	Cytosol /Nucleus	Binding
G4LWI2	Heat shock protein HSP60	0.001447	Cytoplasm	Heat Shock protein
G4LYN4	ADP-ribosylation factor, arf	0.000271	Membrane	Transporter
G4M1M0	DNA polymerase	0.000801	Nucleus	Binding and catalytic
G4V6R4	Putative rab9	6.42E-05	Membrane	Binding
G4V8L4	Putative heat shock protein 70	3.61E-43	Cytosol /Nucleus	Binding/Heat Shock protein
G4V910	Putative heat shock protein 70 (Hsp70)	0.001976	Cytosol /Nucleus	Binding/Heat Shock protein
G4V8L4	Putative heat shock protein 70	2.60E-29	Cytosol /Nucleus	Binding/Heat Shock protein
G4VAC9	Putative uncharacterized protein	0.001085	Unknown	Unknown
G4VAD2	Elongation factor 1-alpha	1.72E-36	Cytoplasm	Binding/GTPase activity
G4VAW0	Serine/threonine kinase	4.72E-05	Nucleus	Binding
G4VB75	cytoplasmic dynein light chain	0.000763	Cytoskeleton	Structural/Motor
G4VB79	Voltage-gated potassium channel, KCNQ	0.001114	Membrane	Transmembrane Transporter
G4VDD2	Eukaryotic translation initiation factor 5A	0.000224	Ribosome	Binding
G4VG19	Phosphoglycerate kinase	1.25E-43	Mitochondria	Kinase
G4VG20	Phosphoglycerate kinase	2.68E-07	Mitochondria	Kinase
G4VGA0	Sodium/potassium-transporting ATPase subunit alpha	0.000264	Membrane	Transporter
G4VH98	Putative fimbrin/plastin	0.002	Unknown	Binding
G4VHN3	ATP synthase subunit beta	7.63E-34	Membrane	Transporter/ Bindng
G4VIM7	Camp-response element binding protein-related	0.000471	Nucleus	Binding
G4VKT8	Putative atp synthase alpha subunit vacuolar	0.000308	Membrane	Transporter/ Binding
G4VLJ0	ATP synthase subunit alpha	0.000104	Membrane	Transporter/ Binding
G4VLJ8	Fidgetin like-1	0.000756	Nucleus	Binding and catalytic
G4VLN5	Putative uncharacterized protein	3.70E-70	Unknown	Binding
G4VLW1	Putative actin	6.14E-19	Membrane	Binding
G4VLW2	Putative actin-1	2.35E-33	Membrane	Binding
G4VM26	Putative zinc finger protein	0.000636	Nucleus	Binding
G4VMG4	Venom allergen-like (VAL) 3 protein	0.001866	Membrane	Unknown
G4VMT3	Adapter-related protein complex 3, beta subunit	0.001906	Membrane	Transporter
G4VP51	Putative ADP,ATP carrier protein	0.000161	Membrane	Structural/ Transporter
G4VPE8	Putative inorganic pyrophosphatase	6.22E-23	Cytoplasm	Binding
G4VPU8	Putative cytoplasmic dynein intermediate chain 2	9.23E-05	Cytoskeleton	Structural/Motor
G4VQ01	Uncharacterized protein	0.00185	Membrane	Unknown
G4VQ58	Phosphopyruvate hydratase	8.38E-06	Cytoplasm	Binding

Venn diagrams were generated to identify proteins unique to each group. Out of the 36 total *Schistosoma* protein groups confidently identified, 5 (15.6%), 4 (12.6%) and 2 (6.3%) proteins were unique to SH, PS and PT group respectively while only 8 proteins (25%) were found common to all study groups (Fig 7A). Heat shock protein 70, elongation factor 1-alpha, camp-response element binding proteins-related, histone H4 and venom allergen-like (VAL) 3 proteins were found to be unique to SH group while tubulin alpha chain, calreticulin autoantigen homolog, heat shock protein HSP 60 and putative ADP,ATP carrier protein were found only in PS group. 2 potential biomarkers unique to the PT group include cytoplasmic dynein light chain and putative actin 1. 13 (36.1%) of the predicted Schistosoma protein were membrane-associated, 8 (22.2%) nuclear based, 4 (11%) cytoplasmic and 3 (8.3%) cytoskeletal and mitochondrial, 1 (2.8%) ribosomal and 3 (8.3%) unknown.

The result of the three independent t-tests performed using LFQ values for “NPS versus SH”, “NPS versus PT” and “NPS versus PS” revealed a total of 54 candidate human protein biomarkers for schistosomiasis and bladder pathology. The proteins are distributed into 43, 8 and 7 for “NPS versus SH” (Table 3), “NPS versus PT” and “NPS versus PS” (Table 4) respectively. 37 and 2 proteins were unique to SH and PT groups, respectively, while none were unique to the PS group (Fig 7B). A search for possible marker overlap across study groups showed that cathepsin B (P07858) was shared by all disease groups; arylsulfatase A (A0A0C4DFZ2) and phosphatidylethanolamine-binding protein 4 (Q96S96) were shared by PS and SH group (Table 5); and PT and SH groups were found to have 4 proteins in common, namely transthyretin (P02766), plasma retinol-binding protein (Q5VY30), phosphatidylcholine-sterol acyltransferase (P04180) and cartilage intermediate layer protein 2 (K7EPJ4). The majority of the human proteins identified were predicted to be membrane associated and perform “binding” molecular activities. The human proteins that were identified as differentially abundant in the SH, PT and PS groups were subjected to pathway analysis using the reactome tool and string DB. Reactome did not identify any statistically significantly overexpressed human pathways in any of the groups, as the majority of identified proteins did not have mapped identifiers. Using string DB, we found that the cellular component ‘extracellular exosomes’ (GO:0070062) was significantly functionally enriched in the SH group compared to that of the NPS group, with a FDR of 1.09e-14. Furthermore, the Kegg pathway ‘lysosome' (ID 04142) was functionally enriched with an FDR of 0.00685. The number of differentially abundant proteins unique to the PT and PS groups was too few to identify functionally enriched pathways or GO terms.

**Table 3:** Identified differentially abundant human proteins and their predicted functions in individuals infected with *Schistosoma haematobium* (SH) vs the NPS group.

Protein ID	Identified Human Protein	PEP Scores	Location	Predicted Functions
Q99519	Sialidase-1	2.68E+08	Membrane	Binding
A0A075B6I5	Ig lambda chain V-I region NIG-64;Ig lambda chain V-I region BL2	9.46E+08	Membrane Associated	Binding/Immune
Q9BQ51	Programmed cell death 1 ligand 2	4.76E+08	Membrane	Binding/Immune
P09467	Fructose-1,6-bisphosphatase 1	4.59E+08	Cytosol/Nucleus	Binding
Q96L35	Receptor protein-tyrosine kinase;Ephrin type-B receptor 4	4.73E+08	Cytoplasm/Mitochondria	Kinases/Binding
Q08174	Protocadherin-1	6.57E+08	Membrane	Binding
P21796	Voltage-dependent anion-selective channel protein 1	4.55E+08	Membrane/Mitochondria	Transport/Binding
A0A087WXM8	Basal cell adhesion molecule	6.27E+08	Membrane Associated	Binding
A0A087X2B5	Basigin	5.07E+08	Membrane	
Q12794	Hyaluronidase-1	3.94E+08	Lysosome	Other Enzymatic
Q13332	Receptor-type tyrosine-protein phosphatase S;Protein-tyrosine-phosphatase	6.35E+08	Membrane	Other Enzymatic
A6NNI4	Tetraspanin;CD9 antigen	4.59E+08	Membrane	Binding/Immune
Q86UN3	Reticulon-4 receptor-like 2	4.69E+08	Membrane	Binding/Immune
P01701	Ig lambda chain V-I region NEW	8.4E+08	Membrane	Binding/Immune
Q99988	Growth/differentiation factor 15	1.17E+09	Exosome	Binding/Immune
P07858	Cathepsin B;Cathepsin B light chain;Cathepsin B heavy chain	1.19E+09	Membrane	Binding
Q15113	Procollagen C-endopeptidase enhancer 1	2.59E+09	Membrane	Binding
P53634	Dipeptidyl peptidase 1;Dipeptidyl peptidase 1 exclusion domain chain;Dipeptidyl peptidase 1 heavy chain;Dipeptidyl peptidase 1 light chain	1.43E+09	Endoplasmic Recticulum/Golgi Apparatus	Binding/Enzymatic
Q96NY8	Nectin-4;Processed poliovirus receptor-related protein 4	1.98E+09	Cytoskeleton/Membrane	Binding
Q9HCN6	Platelet glycoprotein VI	1.91E+09	Membrane	Binding
Q9NZH0	G-protein coupled receptor family C group 5 member B	7.23E+08	Membrane/Nucleus	Binding
O00560	Syntenin-1	1.24E+09	Membrane	Binding
Q92520	Protein FAM3C	9.39E+08	Membrane Associated	Immune
Q8NBJ4	Golgi membrane protein 1	8.67E+08	Membrane Associated	
P04180	Phosphatidylcholine-sterol acyltransferase	1.19E+09	Extracellular	Enzymatic
K7EPJ4	Cartilage intermediate layer protein 2;Cartilage intermediate layer protein 2 C1;Cartilage intermediate layer protein 2 C2	1.04E+09	Membrane	Unknown
P05109	Protein S100-A8;Protein S100-A8, N-terminally processed	1.19E+10	Membrane /cytosol	Binding
O94919	Endonuclease domain-containing 1 protein	1.24E+10	Membrane	Binding/Enzymatic
P02766	Transthyretin	4.96E+09	Cytoplasm	Binding
Q16777	Histone H2A type 2-C;Histone H2A type 2-A	8.63E+09	Nucleus	Binding
A0A087WV17	Osteoclast-associated immunoglobulin-like receptor	5.67E+09	Membrane	Binding/Immune
Q5VY30	Retinol-binding protein 4;Plasma retinol-binding protein(1–182);Plasma retinol-binding protein(1–181);Plasma retinol-binding protein(1–179);Plasma retinol-binding protein(1–176)	2.34E+09	Membrane	Transporter/Immune
H0Y755	Low affinity immunoglobulin gamma Fc region receptor III-A	3.96E+09	Membrane/cytoskeleton	Transporter/Immune
Q8NFZ8	Cell adhesion molecule 4	2.09E+09	Membrane	Binding
A0A087WZR4	Low affinity immunoglobulin gamma Fc region receptor III-B	2.11E+09	Membrane/cytoskeleton	Transporter/Immune
Q9H8L6	Multimerin-2	2.72E+09		
A0A0C4DFZ2	Arylsulfatase A;Arylsulfatase A component B;Arylsulfatase A component C	1.16E+09	Membrane/ER	Binding
F6X2W2	Neuronal growth regulator 1	3.44E+09	Membrane	Transporter/Binding
P09603	Macrophage colony-stimulating factor 1;Processed macrophage colony-stimulating factor 1	6.79E+08	Membrane/Cytoplasm	Binding/Immune
Q96S96	Phosphatidylethanolamine-binding protein 4	1.9E+08	Cytoplasm/Mitochondria	Binding
P31946	14-3-3 protein beta/alpha;14-3-3 protein beta/alpha, N-terminally processed	3.77E+08	Membrane	Binding
A0A087X0D5	Pro-cathepsin H;Cathepsin H mini chain;Cathepsin H;Cathepsin H heavy chain;Cathepsin H light chain	5.06E+08	Cytoplasm/Nucleus	Enzymatic
P09619	Platelet-derived growth factor receptor beta	2.68E+08	Membrane	Binding

SH—Schistosomiasis; PEP—Posterior error probability; Protein IDs are Uniprot accession numbers

**Table 4:** Identified differentially abundant human proteins and their predicted functions from PT and PS groups vs the NPS group.

Protein ID	Identified Human Protein	PEP Scores	Location	Predicted Functions
A0A0G2JM94	Leukocyte-associated immunoglobulin-like receptor 1	1.1E+10	Membrane	Binding/Immune
P02766	Transthyretin	1.24E+10	Membrane	Binding/Immune
Q5VY30	Retinol-binding protein 4;Plasma retinol-binding protein(1–182);Plasma retinol-binding protein(1–181);Plasma retinol-binding protein(1–179);Plasma retinol-binding protein(1–176)	5.67E+09	Cytoplasm	Binding
A0A0C4DFZ2	Arylsulfatase A;Arylsulfatase A component B;Arylsulfatase A component C	2.72E+09	Nucleus	Enzymatic
P07858	Cathepsin B;Cathepsin B light chain;Cathepsin B heavy chain	1.19E+09	Membrane	Binding
P04180	Phosphatidylcholine-sterol acyltransferase	1.19E+09		
K7EPJ4	Cartilage intermediate layer protein 2;Cartilage intermediate layer protein 2 C1;Cartilage intermediate layer protein 2 C2	1.04E+09	Membrane	Unknown
P98160	Basement membrane-specific heparan sulfate proteoglycan core protein;Endorepellin;LG3 peptide	9.34E+10	Membrane	Binding
Q96S96	Phosphatidylethanolamine-binding protein 4	6.79E+08	Membrane Associated/ Lysosome	Binding

PT—Bladder Pathology; PS—Pathology and Schistosomiasis; PEP—Posterior error probability

**Table 5:** Identified differentially abundant human proteins and their predicted functions that were shared between individuals with combined structural bladder pathology and *Schistosoma* infection (PS) and *Schistosoma* infected individuals (SH).

Protein ID	Identified Human Protein	PEP Scores	Location	Predicted Functions
A0A0C4DFZ2	Arylsulfatase A;Arylsulfatase A component B;Arylsulfatase A Component C	2.72E+09	Membrane/ER	Binding
Q96S96	Phosphatidylethanolamine-binding protein 4	6.79E+08	Membrane Associated/ Lysosome	Binding
P07858	Cathepsin B;Cathepsin B light chain;Cathepsin B heavy chain	1.19E+09	Membrane	Binding

PS—Pathology and Schistosomiasis; PEP—Posterior error probability; ID—Identification

## Discussion

A total of over 2,000 proteins are estimated to be present in normal human urine [23], and the highest number of human proteins identified in a proteomic study thus far is 1,823 [24]. In the present study, we observed 1,306 proteins in human urine by unfractionated MS analysis. Sample clustering analyses by PCA and heatmap placed all sample groups into clear-cut strata based on the LFQ intensities of the identified proteins, with little interference between groups, thereby indicating a distinct difference in proteomic signatures between groups.

Some of the potential *Schistosoma* biomarkers identified in this study are clear targets for the generation of new vaccines and drug targets against schistosomiasis. The majority of these proteins appear to be involved in binding activity according to GO-enrichment analysis. This observation is similar to the report of Sotillo *et al*. [16]. The parasite markers include four heat shock proteins (HSPs) which are known as highly conserved stress-induced proteins found in many trematodes and nematodes including *Schistosoma* specific study [25]. HSP expression in the earliest stages of intra-mammalian schistosomula development has been reported and was suggested to be as a result of thermal changes in the parasite niche/environment i.e. changes between freshwater and the human body [16, 26]. Venom allergen-like protein (VAL)-3 was identified in the present study. Sotillo *et al*. [16] reported downregulation of VAL-4 and -6 on the maturing schistosomula tergument and the upregulated expression of VAL-6 in cercaria and adults worm has also been reported [27]. The *Schistosomas* VALs comprise at least 29 members, subdivided into two major groupings, with group 1 including SmVAL1–5, 7–10, 12, 14–15, & 18–29 which have signal peptides and conserved cysteines positioned for disulphide bond formation. VAL proteins are associated with excretion/secretion products and extracellular environment of the parasite [28] and have been used as a trial vaccine against hookworm infections in humans [29]. Changes in the *val* gene and the resultant protein expression denotes its functions in different aspects of host-parasite biology, which include snail invasion by miracidium, intra-molluscan sporocyst development, and cercarial development and host penetration [28]. The VAL protein family are abundant in different helminth species including gastrointestinal nematodes, where they are known to carry out several roles in the infective activities of parasites [16, 30].

Actin 1 protein, as reported in this study, has been identified as a possible drug target for the treatment of schistosomiasis. Strong association between actin and *S*. *mansoni* adult worm surface membranes has been confirmed [25, 31]. Studies have described the role of actin in enhancing the activity of praziquantel (PQZ) treatment of schistosomiasis. It is suggested that PQZ intercalates in the surface membrane lipid bilayers, thereby inducing tegumental changes that leads to antigen exposure, including actin [31, 32].

Elongation factor 1-alpha, phosphopyruvate hydrase and histone-4 were all identified as potential *Schistosoma* biomarkers in this report, which parallels the results of deWalick *et al*. [25], where these proteins were identified in purified eggshell fragments of *Schistosoma mansoni*. The proteins identified as part of the eggshell protein skeleton are known schistosome antigens and may induce cellular or antibody responses [25]. These eggshell markers may be very useful schistosomiasis diagnostic candidates rather than vaccine candidates, since such a vaccine would be likely to target the eggs and further encourage granuloma formation and pathology rather than priming the immune system against the parasite. The significantly regulated parasite proteins were mostly predicted to be membrane-associated, when classified according to their predicted subcellular location [16, 25]. The expression of some membrane associated proteins was earlier proposed as possible vaccine antigens in different *Schistosoma spp* [16, 33]. Jossic *et al*. [34] reported membrane proteins as one of the most interesting classes of proteins among disease biomarker candidates due to their localization on the surface cells and organelles. The identification of a large number of membrane and membrane-associated proteins in the present study strongly suggests that these proteins are abundant in the urine of schistosomiasis patients and would therefore be reasonable targets for drug or vaccine development. These proteins are likely present in the urine as a result of their accessibility to the host immune system via the parasitic tegument, which constitutes the host-parasite interface between *S haematobium* and the human immune system. Despite some recent clarification regarding common transmembrane tegument proteins in *S*. *mansoni* [35], it remains unclear what proteins are abundantly present at this important interface in *S*. *haematobium*. An ideal vaccine candidate would present a large extracellular domain, such as the tetraspanin family of proteins, which are a promising candidate for *S*. *mansoni* vaccines [36]. The present study therefore offers some insight in to potential membrane protein targets for vaccine development in *S*. *haematobium*.

Arylsulfatase A and phosphatidylethanolamine-binding protein 4 were both found in the SH and PS sampled group. Arylsulfatases A, B, and C (arylsulfo-hydrolases) are a group of hydrolytic enzymes that occur in various tissues and fluids [37]. An increase in the activities of arylsulfatase B (ASB) has been reported in bladder tumours [38]. Also, arylsulfatase A (ASA) in the livers of *Schistosoma* infected mice displayed a non- significant decrease in expression vs the control, while the expression of hepatic ASB was significantly increased in *Schistosma* infected mice in similar study [37]. Aminophospholipids, such as phosphatidylserine and phosphatidylethanolamine are described as specific, accessible and stable markers of the luminal surface of tumour blood vessels [39]. There has already been some development of aminophospholipid-targeted diagnostic and therapeutic constructs for use in tumour intervention. Antibody-therapeutic agent conjugates and constructs that bind to aminophospholipids, including methods that specifically deliver therapeutic agents, such as toxins and coagulants, to the constitutively-expressed aminophospholipids of tumour blood vessels, thereby inducing thrombosis, necrosis and tumour regression, are particularly promising [39].

One of the four proteins shared by PT and SH samples, plasma retinol-binding protein (RBP), is a circulating plasma protein produced in the liver and adipose tissue that transports active natural metabolites of Vitamin A as retinol around the body [40]. Retinol acts pharmacologically to restore differentiation and inhibit growth in some premalignant and malignant cell both in vivo and in vitro (including bladder cancer cases) and also modulates cell proliferation, malignant transformation, apoptosis and the immune system [40, 41]. A recent study revealed that individuals with HIV and *S*. *mansoni* coinfection have significantly lower blood RBP levels when compared to participants with HIV and *S*. *haematobium* coinfection [40]. Transthyretin has also been identified by Yi-Ting *et al*., [42] as a potential urine-derived biomarker for bladder cancer.

The programmed cell death 1 (PD-1) surface receptor binds to two ligands, PD-L1 and PD-L2. PD-1–PD-L interaction is known to control the induction and maintenance of peripheral T cell tolerance. PD-1 and its ligands have been exploited by a variety of microorganisms to reduce the effect of antimicrobial immunity, thereby facilitating chronic infection [43]. The findings of Alvarez *et al*. [44] on the role played by PD-1 in innate immunity against *Mycobacterium tuberculosis* also showed that PD-1 signalling might be modulating host innate immunity by inhibiting natural killer (NK) cell responses to the pathogen, contributing to avoidance of immune-mediated pathology caused by excessive host response to the infection. Understanding the functions of PD-1 and its ligands in regulating antimicrobial and self-reactive T cell responses and the possibility of manipulating this pathway may eventually reveal its therapeutic potential in chronic schistosomiasis.

Human Growth/differentiation factor 15 (GDF15) could be a useful diagnostic marker for chronic urinary schistosomiasis. GDF15 is a divergent member of the transforming growth factor β family found in a broad range of cells [45]. Corre *et al*. [45] reported that GDF15 could be an integrative signal in pathological conditions and provide may information on the severity of disease. Expression and secretion of GDF15 is heightened in many malignant tissue and cancer cell lines (prostate, colorectal, pancreatic, gastric and oral squamous carcinoma) as compared with their normal tissues or cells [46, 47, 48, 49, 50, 51, 52].

Human sialidase protein was identified in the SH group and is known for its immunological role in regulating phagocytosis in macrophages cells [53]. Amith *et al*. [54] reported Neu1 sialidase as a complex with Toll like receptor (TLR)-2, -3 and -4, which is induced upon ligand binding to either receptor. Activated Neu1 sialidase hydrolyzes sialyl α-2, 3-linked β-galactosyl residues distant from ligand binding to remove steric hindrance to TLR-4 dimerization, MyD88/TLR4 complex recruitment, NFkB activation and pro-inflammatory cell responses [54].

We identified differentially abundant human proteins in each clinical group that may contribute further specificity to a panel of biomarkers. We found a total of 54 proteins that were differentially abundant compared to the control group, with 43 that were specific for schistosomiasis, 8 that were specific to bladder pathology, and 7 that were specific to those patients with bladder pathology and schistosomiasis. Of these, only 37 and 2 proteins were uniquely identified in the SH and PT groups, respectively, while none were unique to the PS group. This implies that the combined pathology of schistosomiasis and bladder pathology may not have uniquely identifiable characteristics, but rather has features common to both contributing diseases. On the other hand, the proteins that are unique to Schistosoma infected individuals or those with bladder pathology are of interest in identifying the molecular mechanisms underlying the pathology of these diseases and may serve as differential biomarkers for diagnostic purposes. Due to the relatively small numbers of unique differentially abundant proteins identified in each clinical group, pathway analysis is not especially informative. The Reactome database identified no statistically significantly dysregulated pathways, although String analysis identified that ‘extracellular exosomes’ were enriched as a cellular component, as well as the Kegg pathway relating to ‘lysosomes’. This suggests that exosomes may have a role in host/pathogen protein trafficking in the urine, which has been of recent interest in other diseases [55].

This study is currently at the discovery phase of identifying schistosome and human based biomarkers for urinary schistosomiasis and its associated pathologies. Actin 1, elongation factor 1-alpha, phosphopyruvate hydrase, heat shock protein, histone-4 and other schistosome-derived proteins identified in this study could be considered as markers for the diagnosis of urinary schistosomiasis. The presence of venom allergen-like protein-3 in the present study confirms its potential as a promising vaccine biomarker against the parasite. The human programmed cell death 1 and Growth/differentiation factor 15 proteins could also be promising markers for the diagnosis of chronic urinary schistosomiasis, a condition that is difficult to identify by microscopy. The consistency of detection of the human proteins arylsufatase and cathepsin across all disease groups (SH, PT and PS) suggests that these markers may be useful in identifying links between schistosomiasis and the development of urinary bladder cancer. We propose that a panel of biomarkers derived from both human and *Schistosoma* may achieve the best clinical sensitivity and specificity, and this study goes some way to identifying putative candidates for a further quantitative clinical study on a large number of blinded samples.

The reduced complexity of the protein content of urine and its non-invasive sample collection renders urine a valuable source for diagnostic biomarkers, especially for urinary tract diseases. In addition, urine is an ideal biofluid for biomarker discovery by mass spectrometry-based proteomics due to the abundant availability of urine samples and the relative stability of urine proteins. With the use of integrated high throughput technologies, we can begin to elucidate how *S*. *haematobium* and human host systems interact during infection. The momentous challenge we face is the possibility of parasite resistance to the only known drug, Praziquantel, and the ongoing problem of continual re-infection within at-risk populations. The comparative proteomics approach undertaken in this study has generated promising hypotheses regarding the mechanisms of pathogenesis that can be tested through manipulation of the host and parasite during infection. This study demonstrates that urinary proteomics is a viable approach to discovering candidate biomarkers for schistosomiasis and its associated pathology, but the results presented here require validation in a larger cohort before clinical applications can be considered.

## Supporting information

S1 Fig Study workflow showing biomarker discovery experimental design. The study was carried out in two phases (field sampling and laboratory experiments). The abbreviations represent different sample groups namely: SH- *S*. *haematobium* infected groups, PT- bladder pathology group, PS- group with combination of pathology and *S*. *haematobium* infection and NPS- no pathology and schistosomiasis (control group)
(TIF)

S2 Fig The number of peptide sequences identified per sample. Sample names correspond to the following clinical classifications: TC1-15 = PS, TC16-27 = SH, TC28-39 = PT, and TC40-49 = NPS. SH- *S*. *haematobium* infected groups, PT- bladder pathology group, PS- group with combination of pathology and *S*. *haematobium* infection and NPS- no pathology and schistosomiasis (control group)
(TIF)

S3 Fig The number of MS/MS scans identified as a percentage of the total for each sample. Sample names correspond to the following clinical classifications: TC1-15 = PS, TC16-27 = SH, TC28-39 = PT, and TC40-49 = NPS. SH- *S*. *haematobium* infected groups, PT- bladder pathology group, PS- group with combination of pathology and *S*. *haematobium* infection and NPS- no pathology and schistosomiasis (control group)
(TIF)

S4 Fig The number of missed cleavages per peptide across all samples. (TIF)

S5 Fig Volcano plots for the NPS versus SH, NPS versus PT and NPS versus PS comparisons. Permutation-based FDR truncation was set at FDR 0.05.
(PPTX)
