# Supplementary figures and images for: Quantitative label-free proteomic analysis of human urine to identify novel candidate protein biomarkers for schistosomiasis
Source: PLoS Negl Trop Dis. 2017 Nov 8;11(11):e0006045. doi: 10.1371/journal.pntd.0006045 (PMC5695849; doi:10.1371/journal.pntd.0006045)

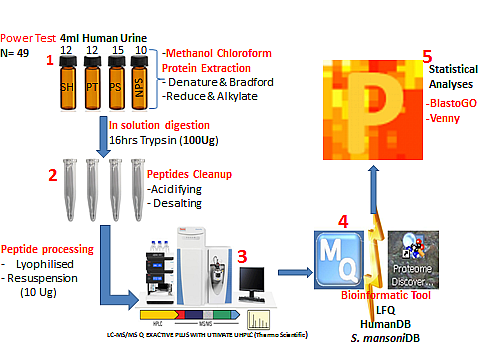

Supplement: S1 Fig — The study was carried out in two phases (field sampling and laboratory experiments). The abbreviations represent different sample groups namely: SH- S. haematobium infected groups, PT- bladder pathology group, PS- group with combination of pathology and S. haematobium infection and NPS- no pathology and schistosomiasis (control group) (TIF) [file pntd.0006045.s001.tif]

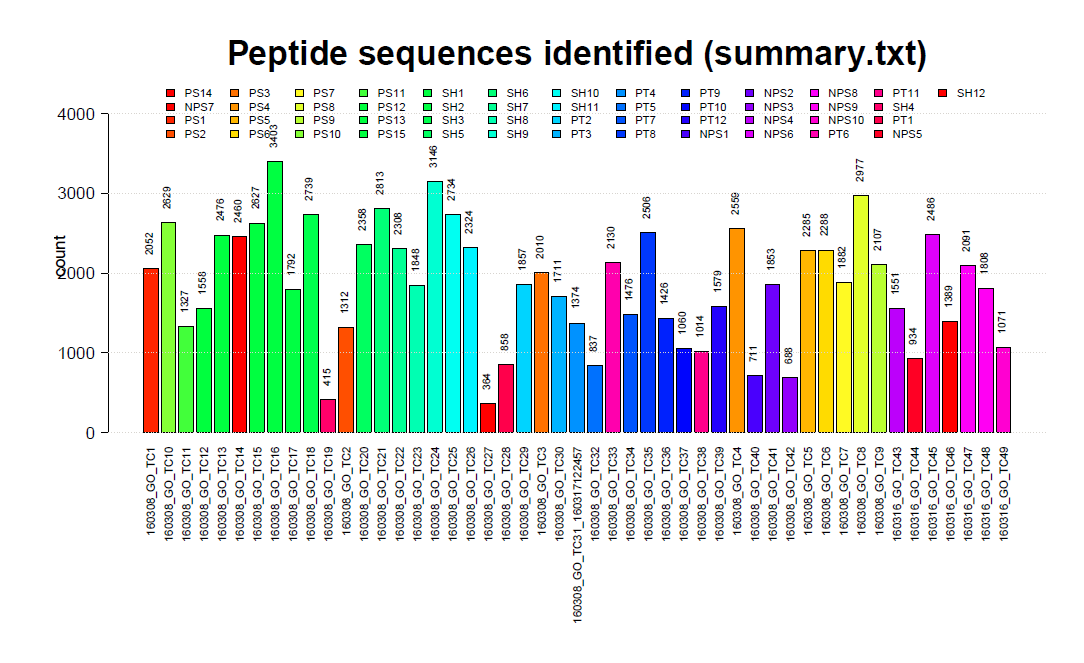

Supplement: S2 Fig — Sample names correspond to the following clinical classifications: TC1-15 = PS, TC16-27 = SH, TC28-39 = PT, and TC40-49 = NPS. SH- S. haematobium infected groups, PT- bladder pathology group, PS- group with combination of pathology and S. haematobium infection and NPS- no pathology and schistosomiasis (control group) (TIF) [file pntd.0006045.s002.tif]

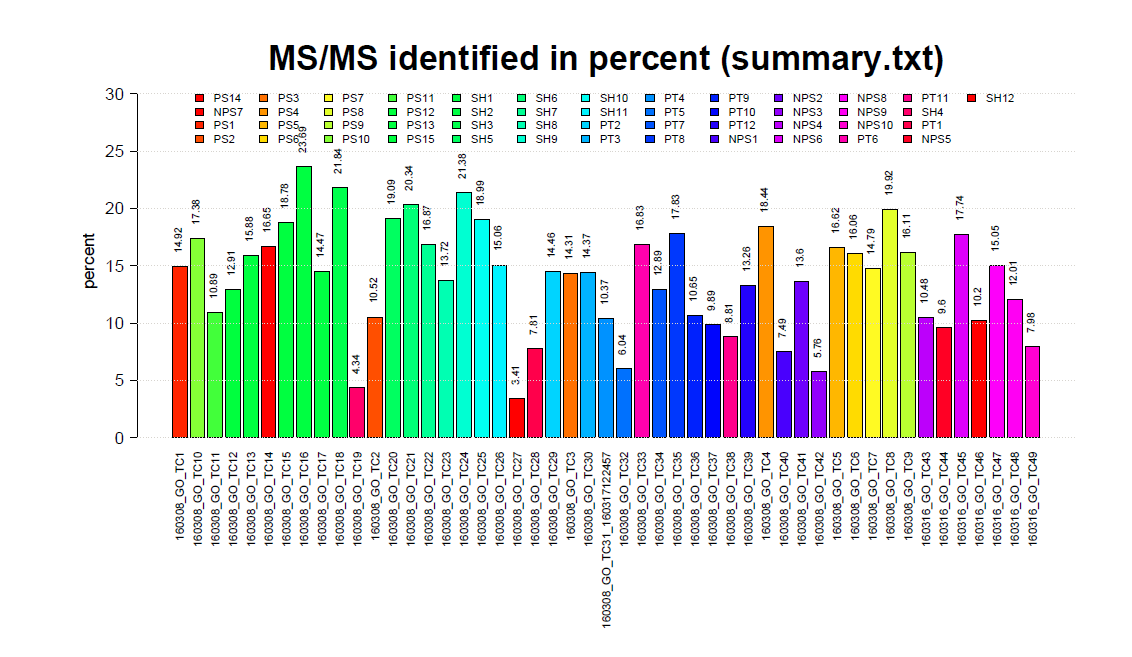

Supplement: S3 Fig — Sample names correspond to the following clinical classifications: TC1-15 = PS, TC16-27 = SH, TC28-39 = PT, and TC40-49 = NPS. SH- S. haematobium infected groups, PT- bladder pathology group, PS- group with combination of pathology and S. haematobium infection and NPS- no pathology and schistosomiasis (control group) (TIF) [file pntd.0006045.s003.tif]

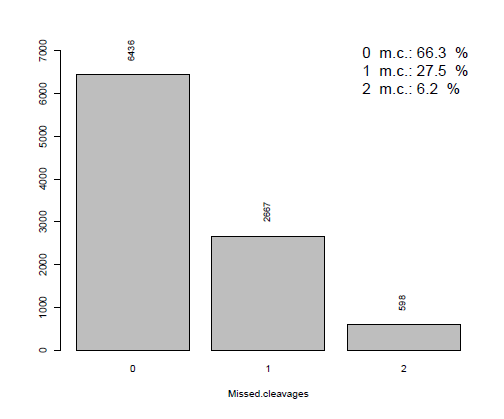

Supplement: S4 Fig — (TIF) [file pntd.0006045.s004.tif]

## Slide 1
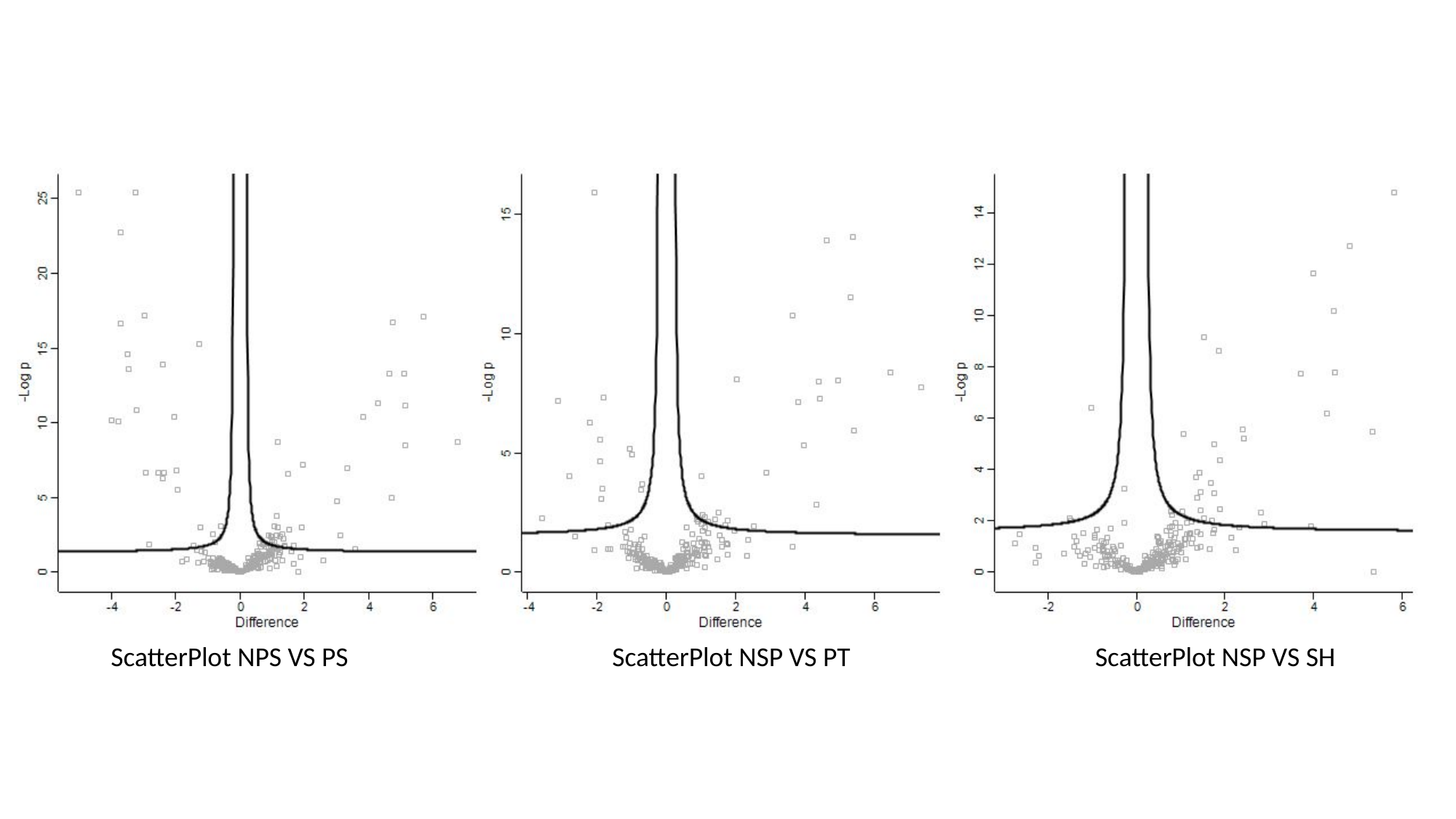

ScatterPlot NPS VS PS
ScatterPlot NSP VS PT
ScatterPlot NSP VS SH

Supplement: S5 Fig — Permutation-based FDR truncation was set at FDR 0.05. (PPTX) [file pntd.0006045.s005.pptx]
